# MntC-Dependent Manganese Transport Is Essential for *Staphylococcus aureus* Oxidative Stress Resistance and Virulence

**DOI:** 10.1128/mSphere.00336-18

**Published:** 2018-07-18

**Authors:** Luke D. Handke, Alexey V. Gribenko, Yekaterina Timofeyeva, Ingrid L. Scully, Annaliesa S. Anderson

**Affiliations:** a Pfizer Vaccine Research and Development, Pearl River, New York, USA; University of Rochester

**Keywords:** MntC, *Staphylococcus aureus*, manganese, manganese transport, virulence factors

## Abstract

Staphylococcus aureus is a human pathogen that has developed several approaches to evade the immune system, including a strategy to resist oxidative killing by phagocytes. This resistance is mediated by production of superoxide dismutase (SOD) enzymes which use manganese as a cofactor. S. aureus encodes two manganese ion transporters, MntABC and MntH, and a possible Nramp family manganese transporter, exemplified by S. aureus N315 SA1432. Their relative contributions to manganese transport have not been well defined in clinically relevant isolates. For this purpose, insertional inactivation mutations were introduced into *mntC*, *mntH*, and SA1432 individually and in combination. *mntC* was necessary for full resistance to methyl viologen, a compound that generates intracellular free radicals. In contrast, strains with an intact *mntH* gene had a minimal increase in resistance that was revealed only in *mntC* strains, and no change was observed upon mutation of SA1432 in strains lacking both *mntC* and *mntH*. Similarly, MntC alone was required for high cellular SOD activity. In addition, *mntC* strains were attenuated in a murine sepsis model. To further link these observations to manganese transport, an S. aureus MntC protein lacking manganese binding activity was designed, expressed, and purified. While circular dichroism experiments demonstrated that the secondary and tertiary structures of this protein were unaltered, a defect in manganese binding was confirmed by isothermal titration calorimetry. Unlike complementation with wild-type *mntC*, introduction of the manganese-binding defective allele into the chromosome of an *mntC* strain did not restore resistance to oxidative stress or virulence. Collectively, these results underscore the importance of MntC-dependent manganese transport in S. aureus oxidative stress resistance and virulence.

**IMPORTANCE** Work outlined in this report demonstrated that MntC-dependent manganese transport is required for S. aureus virulence. These study results support the model that MntC-specific antibodies elicited by a vaccine have the potential to disrupt S. aureus manganese transport and thus abrogate to its virulence.

## Abstract (precis)

Work outlined in this report demonstrated that MntC-dependent manganese transport is required for S. aureus virulence. These study results support the model that MntC-specific antibodies elicited by a vaccine have the potential to disrupt S. aureus manganese transport and thus abrogate to its virulence.

## INTRODUCTION

The acquisition of transition metal ions is essential for all forms of life. It has been estimated that up to a third of all proteins (1) and almost half of all enzymes (2) require a metal cofactor to function. Thus, in an attempt to limit the proliferation of invading bacterial pathogens, the host sequesters transition metal enzyme cofactors, including iron, zinc, and manganese, in a process called nutritional immunity (3, 4). Historically, investigations sought to define how, in the face of limitations imposed by the host, bacterial pathogens acquire iron during infection. However, in recent years, there has been increasing interest in manganese uptake and function.

Numerous biochemical functions for manganese have been described in diverse bacterial species that range from roles in basic physiology, metabolism, signal transduction, and cell division (5–8) to oxidative stress response (9, 10) and virulence (11–16). Given their central role in bacterial physiology and pathogenesis, manganese transporters have been considered targets for therapeutic intervention by active vaccination (17–20) or passive immunization (21) or small-molecule inhibition (22). Accordingly, a growing number of manganese transporters from both Gram-positive and Gram-negative bacteria have now been characterized (6, 13). The manganese transporters from these species belong to either the ATP-binding cassette (ABC) or the Nramp transporter superfamilies (reviewed in references 23 and 24, respectively). Many bacterial species, including Escherichia coli, Streptococcus pyogenes, and Streptococcus pneumoniae, encode only one type of Mn^2+^ transport system, while others, including Bacillus subtilis, Salmonella enterica serovar Typhimurium, Yersinia pestis, and Staphylococcus aureus, encode a member of each transporter family (11, 25–27).

S. aureus is an opportunistic pathogen that places a significant burden on human health: it is the most common etiological agent of skin and soft tissue infections (28, 29) and a significant cause of many other serious diseases, including endocarditis, osteomyelitis, nosocomial pneumonia, and bacteremia (30, 31). The success of this pathogen can be attributed in part to its deployment of multiple mechanisms for immune evasion, including resistance to oxidative stress (32). It has been noted that, despite the presence of high concentrations of toxic reactive oxygen species (ROS) in the phagosome, S. aureus can resist killing by host neutrophils (33, 34). Manganese plays a central role in this resistance through direct detoxification of superoxide radicals and by serving as a cofactor for two superoxide dismutases (SOD), SodA and SodM (9, 27, 35–37).

As described above, S. aureus encodes a member of each manganese transporter family, an ABC transporter, MntABC, and an Nramp transporter, MntH (27). In addition, a second possible Nramp manganese transport protein, annotated as S. aureus SA1432 in strain N315, was identified previously (6). Of these three transport systems, MntABC is the best characterized. In this tripartite transporter system, *mntA* encodes the nucleotide-binding domain, *mntB* encodes the transmembrane domain, and *mntC* encodes the substrate-binding lipoprotein. Mutation of *mntA* (together with the corresponding loss of MntABC transporter function) has been shown to increase sensitivity to methyl viologen (27), a compound that mimics the neutrophil oxidative burst by its generation of intracellular superoxide radicals (38, 39). This heightened sensitivity to methyl viologen has also been demonstrated previously with *mntC* insertional inactivation mutants from a diverse panel of S. aureus isolates (40) as well as after binding of an MntC-specific antigen-binding fragment (Fab) to a wild-type isolate (41). Recently, it was shown that an S. aureus USA300 *mntC* mutant strain was significantly more susceptible to killing by human neutrophils and resumed growth more slowly following exposure to an oxidative burst than its isogenic wild-type counterpart (42). While an early study demonstrated that inactivation of both *mntA* and *mntH* was necessary to significantly reduce recovery of S. aureus laboratory isolate 8325-4 in a murine skin abscess model (27), these findings were confounded by the fact that the strain under investigation may not have had any MntH activity due to a nonsense mutation (43, 44). Later work by Diep et al. showed that a USA300 *mntC* mutant strain was attenuated in a mouse model of sepsis (45). In addition to its role in metal ion transport, S. aureus MntC has been described as a putative adhesin, binding components of the extracellular matrix, a function that may contribute to S. aureus virulence (46).

The relative levels of importance of the S. aureus MntABC and MntH manganese transport systems have not been fully established. In addition, the potential role of SA1432 in manganese transport has not been explored. In the current study, we used a panel of S. aureus mutants to establish their relative levels of importance in oxidative stress resistance. We show that, among these transport systems, the ABC transporter, MntABC, is primarily responsible for resistance to methyl viologen and SOD activity under conditions of low manganese availability and that MntH and SA1432 are not. Further, *mntC* knockout mutant strains were attenuated in a mouse sepsis model. An MntC protein defective in manganese binding was designed, expressed, purified, and characterized. In contrast to complementation with wild-type *mntC*, introduction of this manganese-binding defective allele into the chromosome of an *mntC* strain failed to restore either resistance to methyl viologen or virulence. Taken together, the results presented here show that MntABC-dependent manganese transport is essential for S. aureus oxidative stress resistance and pathogenicity.

(Portions of this work were presented in poster format at the International Conference on Gram-Positive Pathogens, 9 to 12 October 2016, in Omaha, NE, and at the American Society for Microbiology Conference on Antibacterial Development, 11 to 14 December 2016, in Washington, DC.)

## RESULTS

### Identification of S. aureus N315 SA1432, a putative Nramp transporter.

**FIG 1:**
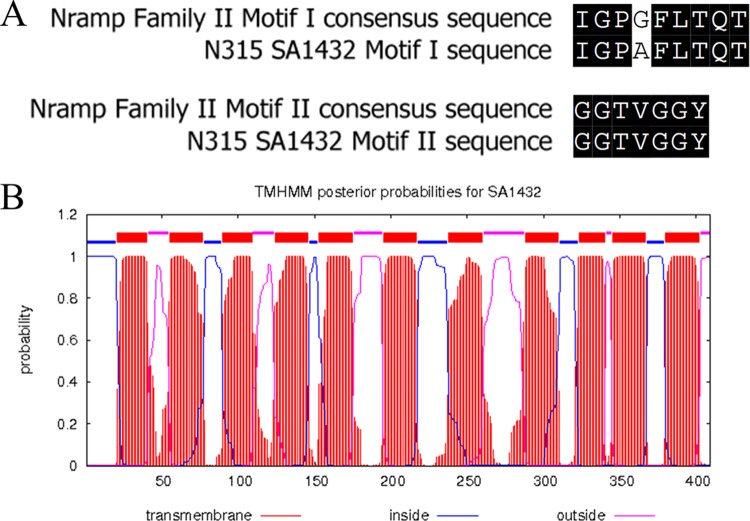
Characterization of S. aureus N315 SA1432. Nramp family II motifs that were previously identified (6) were aligned against motifs present in S. aureus N315 SA1432 (A). Transmembrane helices present in S. aureus N315 SA1432 were predicted with TMHMM v. 2.0 (47) (B). The probability of protein regions being present as transmembrane helices is plotted in red, as cytoplasmic domains in blue, or as extracellular domains in pink.

A previous review of bacterial manganese transporters classified putative Nramp proteins into three families based on the presence of two conserved amino acid motifs unique to each family (6). Analysis of the primary amino acid sequence of S. aureus N315 SA1432 revealed sequences that are highly similar to consensus motif I (IGPGFLTQT; 88.9% identity) and motif II (GGTVGGY; 100% identity) sequences from the family II subclass of Nramp proteins (Fig. 1A). To determine whether SA1432 is organized in a fashion similar to that seen with other Nramp proteins, a prediction of transmembrane regions of SA1432 was performed with TMHMM 2.0 software (47). This analysis showed that SA1432 possesses cytoplasmic N-terminal and extracellular C-terminal domains flanking 11 transmembrane helices (Fig. 1B), a topology similar to that of E. coli MntH (48) and frequently encountered with bacterial Nramp proteins (24, 49). Finally, SA1432 and its homologs from other S. aureus isolates are annotated as Nramp transporters in the TransportDB 2.0 membrane transporter database (50). For these reasons, the potential role of SA1432 in manganese transport, as assessed by oxidative stress resistance and SOD activity, was included in the analysis of MntH and MntABC in the current studies.

### At low concentrations of manganese, MntABC is of primary importance in conferring resistance to oxidative stress and SOD activity.

In order to better establish the relative contributions of manganese transporters to S. aureus oxidative stress resistance, insertional inactivation mutations were made in *mntC*, *mntH*, and SA1432 both singly and in combination. The complete mutant set was made in a laboratory isolate, 8325-4, and in a clinical isolate, PFESA0179 (40). Resistance to oxidative stress was assessed by determination of the MIC of methyl viologen, a compound that generates intracellular superoxide radicals (38, 39). For these experiments, S. aureus strains were cultured in TSB-c, tryptic soy broth that was depleted of polyvalent metal ions and then supplemented with MgCl_2_ and FeSO_4_ to final concentrations of 50 µM and 1 µM, respectively (see Materials and Methods). Consistent with our previous results (40), inactivation of *mntC* resulted in a substantial increase in sensitivity to methyl viologen in both strain backgrounds (125-fold in 8325-4 and 16-fold in PFESA0179; Table 1). Irrespective of the presence or absence of *mntH* or SA1432, all strains with intact *mntC* displayed levels of methyl viologen resistance similar to those displayed by the corresponding wild-type strains, indicating that *mntC* plays a dominant role in this process. In contrast to the 8325-4 strain background, a slight increase in sensitivity, from 1.56 to 0.78 mM, was observed when an *mntH* mutation was introduced into the PFESA0179 *mntC* or PFESA0179 *mntC* SA1432 background. Thus, in PFESA0179, functional *mntH* partially restored oxidative stress resistance. In both strain backgrounds, the methyl viologen MICs of the *mntC mntH* double mutant and *mntC mntH* SA1432 triple mutant were identical, indicating that, under those conditions, SA1432 was dispensable for oxidative stress resistance.

**TABLE 1:** MIC of methyl viologen for wild-type 8325-4 and PFESA0179 and transporter mutant strains

Strain	MV MIC^a^ (mM)	
	8325-4	PFESA0179
S. aureus wild type	25	25
S. aureus *mntC*	0.2	1.56
S. aureus *mntH*	25	25
S. aureus SA1432	25	25
S. aureus *mntC mntH*	0.2	0.78
S. aureus *mntC* SA1432	0.2	1.56
S. aureus *mntH* SA1432	25	25
S. aureus *mntC mntH* SA1432	0.2	0.78

a MV MIC, MIC of methyl viologen.

**FIG 2:**
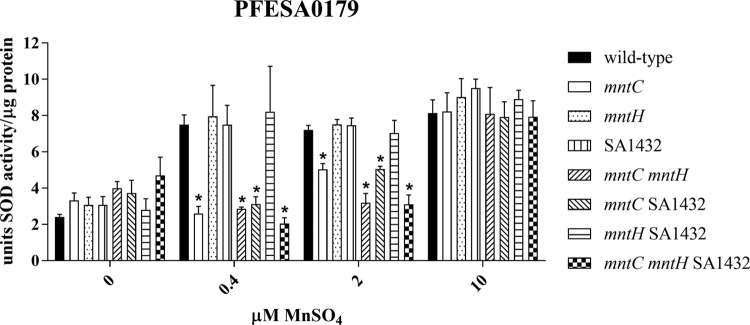
Superoxide dismutase (SOD) activity in wild-type and transporter mutant strains. Wild-type and transporter mutant strains of PFESA0179 were tested for SOD activity following culture in the absence of supplemental manganese or in the presence of 0.4 µM, 2 µM, or 10 µM supplemental manganese. Bars represent means of results from three independent experiments, where SOD activity per microgram protein was assessed in triplicate in each experiment. Error bars represent 1 standard deviation from the mean, and asterisks indicate a statistically significant difference (*P* ≤ 0.001) from the wild-type strain results.

As has been described previously (35, 36), S. aureus encodes two SOD enzymes, SodA and SodM. While SodA enzymatic activity is strictly dependent on manganese as a cofactor, it has been recently shown that SodM can utilize either manganese or iron as a cofactor (37). Therefore, to evaluate the relative contribution of each transport system (MntABC, MntH, and SA1432) to manganese transport as reflected by manganese-dependent SOD activity and to exclude the contribution of Fe-SodM, strains were cultured under iron-replete (1 µM FeSO_4_) conditions. The cells were harvested during the early exponential phase of growth, and SOD activity was assessed for both wild-type and mutant strains of PFESA0179. For these experiments, bacterial cells were cultured in medium without supplemental manganese or in medium supplemented with 0.4 µM, 2 µM, or 10 µM MnSO_4_. Similar low levels of SOD activity were observed across all PFESA0179 strains in medium lacking supplemental manganese (Fig. 2). At the supplemental concentration of 0.4 µM MnSO_4_, a statistically significant increase in SOD activity was observed with the wild-type strain compared with all *mntC* mutant strains, including those with functional *mntH* and/or SA1432. At the concentrations of 0.4 µM and 2 µM MnSO_4_, the differences between the wild-type strain and all other strains with intact *mntC* were not significant. In the PFESA0179 *mntC* and PFESA0179 *mntC* SA1432 strains, an intermediate level of SOD activity was observed at the supplemental MnSO_4_ concentration of 2 µM, indicating that MntH alone could partially restore SOD activity in this background. However, the level of SOD activity conferred by MntH was significantly lower than that of the wild-type strain and lower than those of the other strains encoding intact *mntC*. All strains, including the triple mutant, exhibited similar elevated levels of SOD activity when the medium was supplemented with 10 µM MnSO_4_. This observation could be explained by the presence of an additional transporter with promiscuous manganese transport activity. Notably, the SOD activities of the *mntC mntH* and *mntC mntH* SA1432 strains did not substantially differ at any MnSO_4_ concentration tested, indicating that functional SA1432 is not important for manganese import under those conditions.

Taken together, these results underscore the fact that, under manganese-limited conditions, MntABC is the preeminent manganese transporter in S. aureus and is chiefly responsible for supplying the manganese necessary for oxidative stress resistance and SOD activity *in vitro*. In contrast, MntH appears to play a relatively minor role in manganese transport under these conditions as indicated by its limited contribution to methyl viologen resistance and SOD activity. No contribution to these processes could be assigned to SA1432.

### S. aureus *mntC* mutants are attenuated in a mouse model of sepsis.

**FIG 3:**
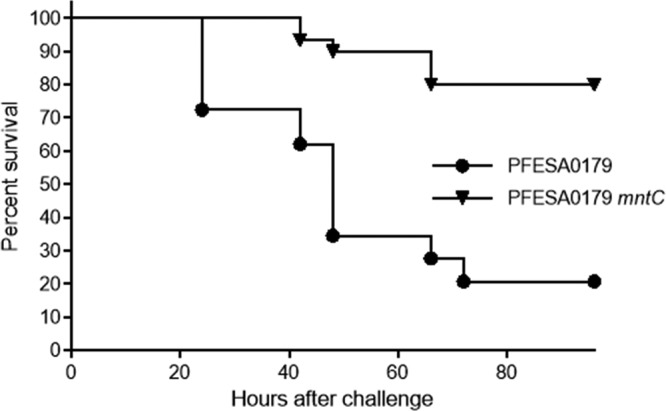
Virulence of PFESA0179 and PFESA0179 *mntC* in a mouse model of sepsis. Survival of mice challenged intravenously with 10^8^ CFU of wild-type PFESA0179 (*n* = 29, circles) or PFESA0179 *mntC* (*n* = 30, triangles, *P* < 0.0001) was monitored for 4 days postchallenge. The figure depicts results from a meta-analysis of three independent experiments performed with 9 or 10 mice per experimental arm.

As described above, MntABC has a primary role in resistance to oxidative stress and SOD activity *in vitro*. To extend these *in vitro* findings and verify that *mntC* mutants display defects in virulence, wild-type PFESA0179 and PFESA0179 *mntC* were tested in a mouse model of sepsis (Fig. 3). For these experiments, groups of CD1 mice were inoculated with ~1 × 10^8^ CFU of each strain by tail vein injection, and survival was monitored for 4 days postchallenge. Relative to the wild-type strain, a statistically significant reduction in virulence was observed in the *mntC* mutant strain (*P* < 0.0001). To confirm that the reduced virulence observed with PFESA0179 *mntC* is not unique to the PFESA0179 strain background, the virulence of another clinical isolate, PFESA0186, and that of its isogenic *mntC* mutant were compared in the mouse sepsis model. In agreement with observations made with PFESA0179 *mntC*, PFESA0186 *mntC* was significantly attenuated compared to its wild-type counterpart (*P* < 0.0001; see Fig. S1 in the supplemental material).

10.1128/mSphere.00336-18.2 FIG S1  Virulence of PFESA0186 and PFESA0186 *mntC* in a mouse model of sepsis. Survival of mice challenged with wild-type PFESA0186 (*n* = 30, line with circles) or PFESA0186 *mntC* (*n* = 29, line with triangles, *P* < 0.0001) was monitored for 5 days postchallenge. The figure depicts results from a meta-analysis of three independent experiments. Download FIG S1, PDF file, 0.3 MB. Copyright © 2018 Handke et al. 2018 Handke et al. This content is distributed under the terms of the Creative Commons Attribution 4.0 International license.

### MntC H50K H123K retains structural integrity but does not bind manganese.

**FIG 4:**
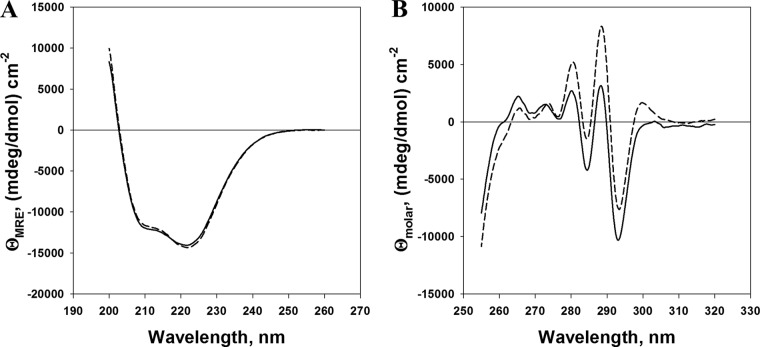
Circular dichroism spectra of wild-type MntC and MntC H50K H153K. Far-UV CD spectra (secondary structure) (A) and near-UV CD spectra (tertiary structure) (B) were generated for the reference strain, wild-type MntC strain (dashed line), and MntC H50K H123K strain (solid line). Spectra were recorded as described in Materials and Methods.

To determine whether the observed attenuation of the PFESA0179 *mntC* mutant in the mouse sepsis model could be directly attributed to defects in manganese transport, a manganese binding-deficient allele of *mntC* was designed for use in complementation studies. The crystal structure of MntC has been solved independently by two groups (41, 51). In both of those studies, side chains from residues H50, H123, E189, and D264 were shown to coordinate manganese binding. Amino acid substitutions H50K and H123K were chosen in order to introduce permanent positive charges at neutral pH in the manganese binding site of MntC, thereby interfering with binding of the positively charged manganese ion while maintaining the overall protein structure. Wild-type MntC and MntC H50K H123K were expressed in E. coli and purified, and the structural integrity of the mutant was confirmed using far- and near-UV circular dichroism (CD) spectroscopy, providing information on the secondary structure and tertiary structure of the protein, respectively (Fig. 4). The far-UV CD spectrum of MntC H50K H123K (Fig. 4A) was identical to that of the wild-type protein, indicating that these amino acid substitutions had no effect on the secondary structure of MntC.

The near-UV CD spectra of MntC H50K H123K and the wild-type protein are shown in Fig. 4B. The overall shape of the spectrum of the mutant is comparable to that of the wild-type strain and suggests that the mutant retains a compactly folded tertiary structure, although differences in the intensities of the spectral bands are apparent. These differences are likely due to the close proximity of W125 and W207 (which would be expected to significantly contribute to the CD signal in the near-UV range) to the manganese binding site. Furthermore, it was previously shown (51) that binding of manganese to wild-type MntC gives rise to the spectral band at 300 nm, as well as to reduced intensity of the 292-nm band and increased intensity of the 287-nm band. This was confirmed in the spectrum of the wild-type protein (Fig. 4B) compared to that of MntC H50K H123K, consistent with the conclusion that wild-type MntC preparations often contain an irreversibly bound metal ion (51). This provides indirect evidence that MntC H50K H123K is unable to bind divalent metal ions, which was confirmed by the isothermal titration calorimetry (ITC) results described in the following section.

**FIG 5:**
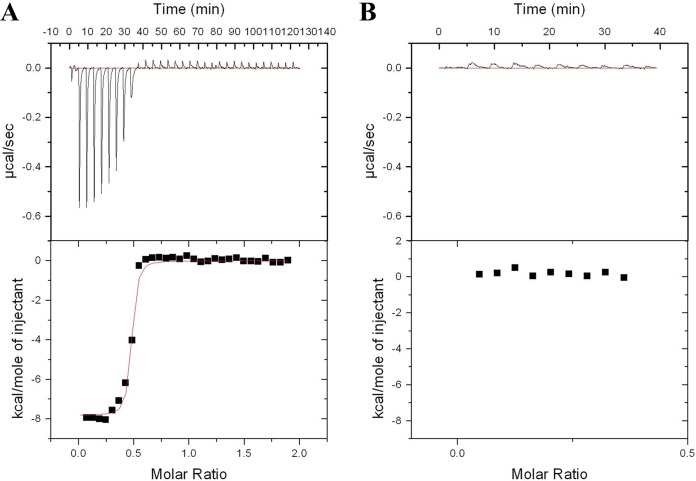
Isothermal titration calorimetry experiments. Mn^2+^ titration was performed with wild-type MntC (A) and MntC H50K H123K (B). The upper panels depict experimental heat flows, and lower panels depict integrated heat flows of each injection plotted as a function of the [Mn^2+^]/[protein] ratio, with the fit of the wild-type MntC data shown as a red line. Binding stoichiometry (N), 0.453 ± 0.004; affinity constant, (2.4 ± 0.8) × 10^7^ M^−1^; enthalpy change upon binding (ΔH), −7.8 ± 0.1 kcal/mol; entropy change upon binding (ΔS), 8.5 (cal/mol) deg^−1^.

Injections of MnCl_2_ solution into a solution of wild-type MntC were accompanied by significant exothermic heat effects as detected by ITC (Fig. 5). As expected, wild-type MntC bound manganese with high affinity. Both the enthalpy and the entropy changes seen upon binding (ΔH and ΔS, respectively) were favorable. While a stoichiometry of one Mn^2+^ ion per MntC protein is expected, the observed partial stoichiometry (0.45 Mn^2+^ ions bound per MntC molecule) can be explained by the presence of an irreversibly bound divalent metal ion. This observation has been made previously with S. aureus MntC, where bound metal ions could not even be extracted by EDTA (51), and is consistent with data determined for the S. pneumoniae manganese transporter component PsaA, which is thought to be irreversibly bound by zinc (52). In contrast to the wild-type MntC protein results, the heat released after MnCl_2_ injection into a solution of MntC H50K H123K was essentially zero and corresponded to the heat released after MnCl_2_ dilution into buffer, indicating that the protein is unable to bind manganese. These ITC results, taken together with the CD data described above, show that amino acid substitutions H50K and H123K indeed eliminated the manganese-binding ability of MntC and yet resulted in no detectable structural changes, as intended.

### An *mntC* strain complemented with MntC H50K H123K is sensitive to oxidative stress.

Integrative complementation vectors were produced to express full-length versions of wild-type MntC or MntC H50K H123K. In these vectors, a 67-bp sequence that resides upstream of the native S. aureus *mntABC* locus was used; this promoter element had been shown in earlier reporter studies to be sufficient to drive expression of *mntC* (40). To prevent potential readthrough expression of the *mntC* alleles, a strong transcriptional terminator (53) was placed upstream of the promoter element in these constructs. In addition to plasmids for expression of wild-type MntC and MntC H50K H123K, a null vector, encoding only the transcriptional terminator and promoter element, was constructed. Each of the complementation plasmids was integrated as a single copy at the *geh* locus in the chromosome of PFESA0179 *mntC* as described previously (40, 54). Expression of wild-type MntC and MntC H50K H123K was subsequently confirmed by Western blotting with an MntC-specific monoclonal antibody (MAb) (Fig. S2).

10.1128/mSphere.00336-18.3 FIG S2  Western blot detection of MntC expression in PFESA0179 *mntC* complementation strains. A Coomassie-stained gel is shown in panel A, and a Western blot probed with MntC-specific MAb 305-78-7 is shown in panel B. Lanes: M, protein molecular weight standard; 1, PFESA0179; 2, PFESA0179 *mntC*; 3, PFESA0179 *mntC* complemented with wild-type MntC; 4, PFESA0179 *mntC* complemented with MntC H50K H123K; 5, PFESA0179 *mntC* complemented with the null vector. Download FIG S2, PDF file, 0.1 MB. Copyright © 2018 Handke et al. 2018 Handke et al. This content is distributed under the terms of the Creative Commons Attribution 4.0 International license.

As expected, introduction of the null vector at *geh* did not restore resistance to oxidative stress in the *mntC* mutant (Table 2). In contrast, integration of a vector expressing wild-type MntC resulted in full restoration of oxidative stress resistance to PFESA0179 *mntC*, as reflected by the unchanged MICs of methyl viologen compared to those seen with wild-type PFESA0179. Consistent with its observed inability to bind manganese *in vitro*, MntC H50K H123K failed to complement the *mntC* mutation and restore methyl viologen resistance. The observed methyl viologen MICs seen with the strains cultured in the presence of 10 µM manganese, a concentration that gave elevated SOD activity in all strains within the panel of transporter mutant strains, were identical (Fig. 2). Collectively, these data confirm the inability of MntC H50K H123K to bind manganese and further tie MntC-dependent manganese transport to susceptibility to oxidative stress.

**TABLE 2:** MIC of methyl viologen for wild-type PFESA0179 and PFESA0179 *mntC* complementation strains

Strain	*mntC* allele used in complementation	MV MIC^a^ (mM)	MV MIC (mM)^a^ + Mn^b^
S. aureus PFESA0179	NA^c^	25	25
S. aureus PFESA0179 *mntC*	Wild type	25	25
S. aureus PFESA0179 *mntC*	H50K H123K	0.78	25
S. aureus PFESA0179 *mntC*	None (null vector)	0.78	25

a MV MIC, MIC of methyl viologen.

b + Mn, medium supplemented with 10 µM MnSO_4_.

c NA, not applicable.

### An *mntC* strain complemented with MntC H50K H123K is attenuated in a mouse sepsis model.

**FIG 6:**
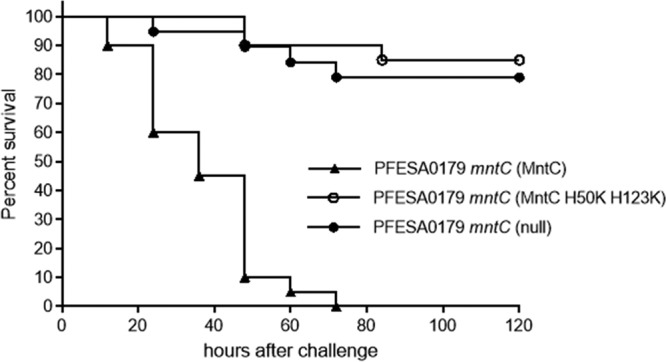
Virulence of PFESA0179 *mntC* complementation strains in a mouse model of sepsis. Survival of mice challenged intravenously with 10^8^ CFU of PFESA0179 *mntC* complemented with wild-type MntC (*n* = 20, triangles), PFESA0179 *mntC* complemented with MntC H50K H123K (*n* = 20, open circles, *P* < 0.0001), or PFESA0179 *mntC* complemented with the null vector (*n* = 19, closed circles, *P* < 0.0001) was monitored for 5 days postchallenge. The figure depicts results from a meta-analysis of two independent experiments performed with 9 or 10 mice per experimental arm.

To determine whether the virulence defects observed with *mntC* mutants are directly linked to manganese transport, the complementation strains expressing wild-type MntC and MntC H50K H123K were tested in the mouse sepsis model. Results from a meta-analysis of two independent experiments are shown in Fig. 6. Complementation with a wild-type copy of *mntC* restored the virulence of PFESA0179 *mntC*, while the strain harboring the null vector was attenuated (*P* < 0.0001). Similarly, compared to the strain encoding wild-type MntC, complementation with MntC H50K H123K did not restore virulence to PFESA0179 *mntC* (*P* < 0.0001). These results confirm that MntC-dependent manganese transport is critical for S. aureus pathogenesis.

## DISCUSSION

Manganese import is required for the virulence of many bacterial pathogens. Fittingly, in a process called “nutritional immunity” (3), the host employs a number of mechanisms to limit the availability of free nutrients, including manganese (25). Critical to manganese restriction in neutrophils is the presence of the protein calprotectin, which is capable of binding manganese and zinc with high affinity (55). The presence of calprotectin has been shown to limit the growth of S. aureus *in vitro* and to enhance its sensitivity to oxidative stress via a reduction in bacterial SOD activity (56). Additional published work performed with S. aureus manganese transporter mutants demonstrated that, when the level of free manganese was partially limited by the presence of calprotectin (at 240 µg/ml), both *mntC* and *mntH* were required for full SOD activity (57). As the level of manganese became further limited by the presence of calprotectin (at 480 µg/ml), SOD activity was significantly diminished in both the Δ*mntC* and Δ*mntC* Δ*mntH* strains but not in the Δ*mntH* strain. Finally, at the highest concentration of calprotectin tested (960 µg/ml) by those investigators, all strains exhibited low SOD activity. These results can be interpreted to mean that MntABC is of greater importance than MntH for SOD activity upon increasing the level of manganese limitation. Results from the SOD assay presented in our work agree with these prior studies, although the approach used was different. In contrast to increasing the level of manganese limitation by calprotectin sequestration, for the current work, known concentrations of manganese were added to the manganese-depleted culture medium prior to SOD activity assessment. Supplementation of the medium with submicromolar manganese concentrations was sufficient to elevate SOD activity but did so only in strains where *mntC* was intact. The notion of MntC, as part of the tripartite MntABC transporter, acting as the main contributor to SOD activity under conditions of manganese limitation is consistent with its reported high affinity for manganese (dissociation constant [*K*_*d*_] in the nanomolar range) (Fig. 5) (51). In contrast, the contribution of MntH to SOD activity was observed only in the absence of *mntC* and only at micromolar manganese concentrations.

A similar set of observations was made when the mutant set was assayed for methyl viologen resistance, where MntC was the primary contributor to oxidative stress resistance, and a minimal contribution and no contribution to resistance could be attributed to MntH and SA1432, respectively. It should be noted that this small contribution of MntH was observed only in the PFESA0179 strain background. This observed difference between PFESA0179 and 8325-4 can be explained by the presence of a preexisting *mntH* nonsense mutation in the 8325-4 strain background (43, 44), a lesion which had not been described when the 8325-4 manganese transporter mutant set was originally constructed. In contrast, sequence analysis of PFESA0179 confirmed the absence of such mutations in *mntC*, *mntH*, and SA1432 (data not shown).

Prior work has suggested that the MntH Nramp transporter is constitutively expressed in S. aureus (27). In contrast, MntABC expression is subject to regulation by MntR, whose repressing activity was shown to be manganese dependent (27). Later work with a luciferase reporter under the control of the *mntABC* promoter showed that MntR-dependent repression is very sensitive, occurring at nanomolar concentrations of manganese (40). Thus, under conditions where manganese is freely available, it is suggested that MntH represents the primary means of manganese acquisition by S. aureus. However, when manganese becomes scarce, MntR-based repression of *mntABC* is relieved. Under these conditions, the high-affinity MntABC transporter is likely the more important contributor to maintenance of a superoxide defense, a concept supported by results from the SOD assay and methyl viologen resistance assessments presented here. Notably, the SOD activity attributable to MntH in the absence of intact *mntC* was significantly lower than that of the wild-type isolate (Fig. 2), indicating that MntH is not fully redundant for the manganese import activity of MntABC. MntC expression is also upregulated in the manganese-limited host environment. In a murine bacteremia model, MntC expression was detectable by immunofluorescence microscopy with a diverse panel of 10 S. aureus isolates (17). This expression was detected in most strains as early as 1 h postinfection and was detectable in all strains at 4 h postinfection (17).

Although the initial characterization work whose results are shown in Fig. 1 favored the possibility that SA1432 was a manganese transporter, results from methyl viologen resistance determinations and the SOD assay do not support this notion. Although the protein may not be involved in manganese acquisition, the level of conservation of SA1432 among diverse isolates of S. aureus argues that it is of some importance to the cell. When 20 isolates from five clinically relevant sequence types (ST) were randomly selected from the internal strain collection of Pfizer, Inc., and aligned against the N315 SA1432 sequence, SA1432 homologs were found in each of the strains (L. D. Handke, unpublished observation). The level of nucleotide identity of SA1432 homologs in these isolates was very high: ST5 strains had an average nucleotide identity of 100%, ST8 strains had an average nucleotide identity of 99.9% (range, 99.2 to 100%), ST22 strains had an average nucleotide identity of 97.5% (range, 97.4 to 97.5%), ST30 strains had an average nucleotide identity of 97.6% (range, 97.5 to 97.6%), and ST45 strains had an average nucleotide identity of 96.3% (range, 96.2 to 96.3%; Handke, unpublished). In addition, all 100 isolates were predicted to encode a fully intact open reading frame (ORF) (Handke, unpublished). Additional work will be required to define the function of this highly conserved protein in S. aureus.

In this report, we demonstrated that S. aureus *mntC* mutant strains are significantly attenuated in animal models of infection. This observation is in good agreement with those of Diep et al., who demonstrated that an S. aureus USA300 *mntC* mutant was significantly attenuated in a mouse lethal challenge model (45). A subsequent report showed that MntC has adhesin activity, an attribute which may contribute to S. aureus pathogenesis (46). As a result, it could not be concluded that the virulence defect in the PFESA0179 *mntC* strain is attributable to defects in manganese transport. Thus, in the current work, an MntC protein that could no longer bind manganese was designed and expressed. The intended inability of this protein variant, MntC H50K H123K, to bind manganese was subsequently confirmed by a biophysical method, i.e., ITC, as well as with a biological assay, i.e., by its failure to restore resistance to methyl viologen. As MntC H50K H123K retained proper secondary and tertiary protein structures as assessed by far-UV and near-UV CD, it should retain its reported adhesin activity. However, complementation of the *mntC* mutant with MntC H50K H123K did not restore virulence in the model. These results indicate that, despite any potential role of MntC in adherence to the extracellular matrix, elimination of the manganese-binding activity of MntC is sufficient to significantly attenuate S. aureus.

MntC is a component of a tetravalent S. aureus vaccine under investigation in clinical trials (ClinicalTrials registration no. NCT02388165). MntC is attractive as a vaccine candidate because of its high level of conservation (17), its expression early during the course of infection (17), and its presence on the cell surface (17, 21, 40, 41). In preclinical studies, active vaccination with purified, recombinant MntC significantly reduced the S. aureus bacterial burden in a mouse model of bacteremia, further supporting the notion that it may be an effective vaccine antigen for the prevention of S. aureus infection (17). MntC has been shown to be highly immunogenic, eliciting a strong antibody response(s) during diverse S. aureus infection states (58–61) and during vaccine clinical trials (62–64).

**FIG 7:**
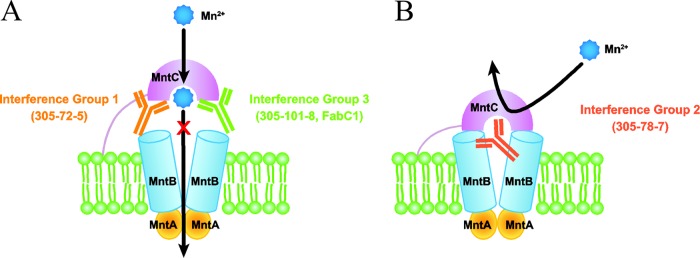
MntC-specific MAbs disrupt MntC function by distinct mechanisms. Mechanisms of activity of MntC-specific MAbs from interference groups 1, 2, and 3 (17) include interference with the interaction of MntB and MntC (A) and direct blockage of manganese binding by MntC (B). The model was adapted from data presented previously (41, 65).

Antibodies against MntC may prevent S. aureus infection in various ways. An MntC-specific antigen-binding fragment (Fab), FabC1, was recently identified by phage display methods (41). As described above, binding of this Fab was shown to enhance the sensitivity of S. aureus to oxidative stress (41). Based on the location of the epitope in the MntC crystal structure, FabC1 binding was predicted to impede manganese transport by disrupting the interaction of MntB and MntC (41). In a separate study, interference mapping of 23 MntC-specific monoclonal antibodies (MAbs) by surface plasmon resonance subdivided these MAbs into three groups, termed interference groups 1, 2, and 3, based on their ability to bind MntC simultaneously (17). Recently, Gribenko et al. suggested a mechanistic explanation of how each of these three groups of MAbs interferes with Mn^2+^ binding (65). Members of interference group 1, which includes MAb 305-72-5, and interference group 3, which includes MAb 305-101-8 (and is predicted to include FabC1 based on detection of overlapping epitopes), interfere with MntB-MntC interaction by binding to two separate lobes of MntC (Fig. 7A). Members of interference group 2, which includes MAb 305-78-7, directly block binding of manganese by MntC (Fig. 7B).

In this study, we have demonstrated that loss of *mntC* by mutation in general and loss of MntC-dependent manganese binding, specifically, attenuated S. aureus in an infection model. Thus, disruption of MntC-dependent manganese transport by antibody binding by the mechanisms outlined above should also attenuate S. aureus. In support of this possibility, passive immunization with two MAbs with distinct MntC-binding activities, MAb 305-78-7 (interference group 2) and MAb 305-101-8 (interference group 3), significantly reduced the S. aureus bacterial burden in an infant rat model of infection (17). Importantly, under the conditions tested, we demonstrated that manganese import by other potential transport systems was either minimal, occurring only at very high concentrations of manganese (MntH), or not observed at all (SA1432). Therefore, under conditions of manganese limitation imposed by the host, S. aureus likely could not bypass antibody-based disruption of MntC function by any of the alternate manganese transport mechanisms that have been identified to date. We conclude that MntC-dependent manganese transport plays a central role in the ability of S. aureus to cause invasive disease, and the current work clearly identifies MntC as a critical virulence factor for this pathogen.

## MATERIALS AND METHODS

### Primers, strains, plasmids, and growth media.

Primers, strains, and plasmids used in the current study are listed in Table S1 in the supplemental material. All primers for PCR were purchased from Integrated DNA Technologies, Inc. (Coralville, IA). S. aureus was propagated in Bacto tryptic soy broth (TSB; BD, Sparks, MD) or on Difco tryptic soy agar (TSA; BD). To make TSB-c medium (40), TSB was stirred in the presence of 1% (wt/vol) Chelex 100 beads (Sigma-Aldrich, St. Louis, MO) for 4 h, a treatment that has been shown to reduce the level of available manganese to the low nanomolar range (40). The medium was then filter sterilized and supplemented with MgCl_2_ and FeSO_4_ to final concentrations of 50 µM and 1 µM, respectively. All antibiotics were purchased from Sigma-Aldrich and were used at the following concentrations for S. aureus: 5 µg/ml (gentamicin), 10 µg/ml (erythromycin), and 7 µg/ml (chloramphenicol).

10.1128/mSphere.00336-18.4 TABLE S1  Strains and reagents used in the study. Download TABLE S1, DOCX file, 0.03 MB. Copyright © 2018 Handke et al. 2018 Handke et al. This content is distributed under the terms of the Creative Commons Attribution 4.0 International license.

### Transmembrane helix prediction and motif alignment.

Prediction of transmembrane helices was performed with TMHMM v. 2.0 on the CBS web server (http://www.cbs.dtu.dk/services/TMHMM/). TransportDB 2.0 (http://www.membranetransport.org/transportDB2/index.html) was used to classify SA1432 as a member of the Nramp family of transporters. Alignment of amino acid motifs was performed with AlignX (Thermo Fisher Scientific, Waltham, MA).

### Construction of S. aureus transporter knockout strains.

An *mntC*::*aacA-aphD* knockout cassette consisting of two fragments homologous to the S. aureus chromosome (*mntC-1* and *mntC-2*), flanking gentamicin resistance genes *aacA* to *aphD*, was constructed as follows. The primer sets (DNA templates) used to amplify each of the fragments were as follows: (i) for *mntC-1*, oLH81/oLH342 (S. aureus strain COL genomic DNA); (ii) for *aacA to aphD*, oLH343/oLH344 (pGO1); (iii) for *mntC-2*, oLH345/oLH314 (COL genomic DNA). All PCRs were performed with iProof polymerase (Bio-Rad, Hercules, CA), and all cloning and molecular biology techniques were performed with enzymes from New England Biolabs (Ipswich, MA) and as described previously (66). After purification, the three PCR products were spliced together by amplification with oLH81 and oLH314, and the knockout cassette was cloned into pSPT181 at the BamHI/XmaI sites to give pLH57. After electroporation into S. aureus RN4220, the plasmid was integrated at *mntC*, and the final *mntC*::*aacA-aphD* mutant was obtained after plasmid excision by secondary recombination. The mutation was introduced into other S. aureus isolates by transduction performed with protocols described previously (67). In the resulting gentamicin-resistant transductants, insertional inactivation of *mntC* was confirmed by PCR.

An *mntH*::*ermC* knockout cassette consisting of two fragments homologous to the S. aureus chromosome (*mntH-1* and *mntH-2*), flanking the erythromycin resistance gene (*ermC*), was constructed as follows. The primer sets (DNA templates) used to amplify each of the fragments were as follows: (i) for *mntH-1*, oLH315/oLH316 (Newman genomic DNA); (ii) for *ermC*, oLH317/oLH318 (pE194); (iii) for *mntH-2*, oLH319/oLH320 (Newman genomic DNA). After purification, the three PCR products were spliced together by amplification with oLH315 and oLH320, and the knockout cassette was cloned into pSPT181 at the BamHI/XmaI sites to give pLH58. After electroporation into S. aureus RN4220, the plasmid was integrated at *mntH*, and the final *mntH*::*ermC* mutant was obtained after plasmid excision by secondary recombination. The mutation was introduced into other S. aureus isolates by transduction performed with protocols described previously (67). In the resulting erythromycin-resistant transductants, insertional inactivation of *mntH* was confirmed by PCR.

A SA1432::*cat* knockout cassette consisting of two fragments homologous to the S. aureus chromosome (SA1432-1 and SA1432-2), flanking the chloramphenicol resistance gene (*cat*), was constructed as follows. The primer sets (DNA templates) used to amplify each of the fragments were as follows: (i) for SA1432-1, oLH321/oLH322 (Newman genomic DNA); (ii) for *cat*, oLH323/oLH324 (pC194); (iii) for SA1432-2, oLH325/oLH326 (Newman genomic DNA). After purification, the three PCR products were spliced together by amplification with oLH355 and oLH356, and the knockout cassette was cloned into pSPT181 at the BamHI/XmaI sites to give pLH59. After electroporation into S. aureus RN4220, the plasmid was integrated at SA1432, and the final SA1432::*cat* mutant was obtained after plasmid excision by secondary recombination. The mutation was introduced into other S. aureus isolates by transduction with protocols described previously (67). In the resulting chloramphenicol-resistant transductants, insertional inactivation of SA1432 was confirmed by PCR.

### Methyl viologen MIC determination.

The MIC of methyl viologen (Sigma-Aldrich) in TSB-c and TSB-c supplemented with 10 µM MnSO_4_ was determined according to standard CLSI guidelines (68) as described previously (40). The stock solution of methyl viologen (4 M in water) was prepared immediately before use. MIC determinations were made during at least three independent assays.

### Superoxide dismutase (SOD) assay.

S. aureus strains were inoculated into 5 ml TSB-c medium and incubated overnight at 37°C with shaking (225 rpm). A volume of 150 µl of overnight cultures was inoculated into baffled 125-ml flasks containing 25 ml of TSB-c and, where indicated, supplemental concentrations of MnSO_4_. Flasks were incubated at 37°C with shaking (225 rpm). Cultures were allowed to grow until they reached an optical density at 600 nm (OD_600_) of 0.6 to 0.9, corresponding to the exponential phase of growth. At that point, 5 ml from each culture was decanted into three 15-ml conical tubes (for triplicate assessment of SOD activity). Cells were pelleted by centrifugation at 2,400 × *g* for 10 min at 4°C. From that point onward, cell pellets were kept on ice. The culture supernatant was removed by aspiration, the pellet was resuspended in 500 µl of ice-cold 1× phosphate-buffered saline (PBS; Mediatech, Corning, NY), and the cells were transferred to microcentrifuge tubes. The cells were pelleted by centrifugation for 2 min at 16,000 × *g* in a microcentrifuge. Following aspiration of the supernatant, the cells were resuspended in 500 µl of ice-cold 1× PBS. Cell suspensions were added to a lysing matrix B tube (MP Biomedicals, Solon, OH), and samples were processed in a Qiagen Retsch MM300 TissueLyser instrument (Qiagen, Valencia, CA) for 60 s at a frequency setting of 30. Tubes were chilled on ice for 2 min. The cell samples were again processed using a TissueLyser instrument as described above. Samples were spun at 16,000 × *g* for 1 min in a microcentrifuge to precipitate cellular debris. A volume of 200 µl of supernatant was extracted and placed in a clean tube, and protein samples were placed on ice. Protein concentrations were determined for each sample in triplicate with a Quick Start Bradford protein assay kit (Bio-Rad). SOD activity was assessed with a SOD assay kit (Sigma-Aldrich) and was determined in triplicate for each strain during at least three independent experiments. Statistically significant differences in the levels of SOD activity in the wild-type and mutant strains were determined with a *t* test performed using GraphPad Prism version 7.04 for Windows (GraphPad Software, Inc., La Jolla, CA).

### Cloning and expression of recombinant MntC.

pLP1215, a vector for expression of recombinant MntC in which the entire N-terminal lipoprotein signal sequence (including the lipobox Cys residue) has been deleted, has been described previously (17). H50K H123K substitutions (where the residue numbering corresponds to the protein lacking the N-terminal lipoprotein signal sequence, identified as residues 1 to 18) designed to abrogate manganese binding were introduced into pLP1215 with a QuikChange Lightning multisite-directed mutagenesis kit (Agilent Technologies, Santa Clara, CA). Mutagenic primers oLH551 (H50K) and oLH552 (H123K) were designed with the Agilent QuikChange Primer Design Tool, and the mutagenesis reaction was performed according to the kit manufacturer’s recommendations. Following sequence confirmation, the resulting clone for expression of MntC H50K H123K was renamed pLH89. Recombinant MntC proteins were expressed in E. coli and purified as described previously (17); however, the hydrophobic interaction chromatography step was omitted in the current work.

### Circular dichroism (CD).

All CD experiments were done on a Jasco J-810 automated recording spectropolarimeter (Jasco, Easton, MD) equipped with a Jasco Peltier-type PTC-423S 6-position cell holder. Temperature was maintained at 20°C. All spectra were recorded with a data pitch of 0.1 nm, a spectral bandwidth of 3 nm, and a scanning speed of 50 nm/min. Data corresponding to five accumulations were collected and averaged for each spectrum. Near-UV CD spectra of the samples at 0.8 to 0.9 mg/ml in a mixture of 50 mM Na cacodylate and 150 mM NaCl (pH 7.0) were recorded between 320 and 250 nm using 1-cm-path-length rectangular quartz cuvettes. Far-UV CD spectra of the samples at 0.12 to 0.13 mg/ml in 1× PBS (pH 7.4) were recorded between 260 and 200 nm using 1-mm-path-length rectangular quartz cuvettes. Far- and near-UV CD spectra of the corresponding buffers were recorded with the same parameters and subtracted from the protein spectra to provide baseline correction. Baseline corrected spectra were smoothed using adjacent-neighbor averaging of 21 points and normalized to either molar (near-UV CD) or mean residue (far-UV CD) ellipticity, using equation 1 or equation 2, respectively, as

(1) $$\Theta_{\text{molar}}=\frac{\Theta_{\text{measured}}}{10\times l\times C}$$

where Θ_molar_ represents the calculated molar ellipticity (in millidegrees per decimole per square centimeter), Θ_measured_ represents the experimentally measured CD signal, *l* represents the cuvette path length (1 cm), and *C* represents the molar concentration of the protein, and

(2) $$\Theta_{\text{MRE}}=\frac{\Theta_{\text{measured}}\times \text{MRW}}{10\times l\times c}$$

where Θ_MRE_ represents the calculated mean residue ellipticity, MRW represents the mean residue molecular weight (113 g/mol), *l* represents the cuvette path length (0.1 cm), and *c* represents the protein concentration in milligrams per milliliter.

### Isothermal titration calorimetry (ITC).

All ITC experiments were done using a VP-ITC isothermal titrating calorimeter (Microcal, Northampton, MA) in a mixture of 50 mM Na cacodylate and 150 mM NaCl (pH 7.0). Proteins were extensively dialyzed against experimental buffer. Wild-type MntC (32.5 µM) or MntC H50K H123K (49.2 µM) samples were titrated with 0.33 mM MnCl_2_ dissolved in the same buffer. The experimental temperature was 37°C. An initial 2-µl injection was followed by 8-µl injections to reach saturation. Heat flows of MnCl_2_ dilution were taken into account by performing buffer titration with the same ligand solution and subtracting the result from the protein titration data. Wild-type MntC titration data were fitted to the data corresponding to the “single class of binding sites” using Origin 5.0 software provided by the ITC manufacturer. No fitting was possible in the case of MntC H50K H123K, since no binding was taking place.

### Cloning and integration of *mntC* complementation alleles.

Integrative vectors for use in expression of wild-type MntC or MntC H50K H123K full-length proteins were constructed as follows. The 5′ portion of *mntC* (including the N-terminal lipoprotein signal sequence) was amplified from S. aureus COL genomic DNA with primers oLH646 and oLH647. The 3′ portion of *mntC* was amplified from either pLP1215 or pLH89 with primers oLH648 and oLH615. After purification, the *mntC* PCR products were spliced together by amplification with oLH646 and oLH615. Following addition of 3′ A overhangs, the spliced *mntC* PCR products were cloned into pCR2.1-TOPO by the use of a TOPO TA cloning kit (Thermo Fisher Scientific). The wild-type *mntC* allele and *mntC* allele encoding H50K H123K substitutions were amplified from these clones with primers oLH651 and oLH652, and a cassette containing the native *mntABC* promoter preceded by a transcriptional terminator (TT-P_*mntABC*_) was amplified from pLH76 (40) with primers oLH649 and oLH650. Integrative vector pLH71 was linearized with a SmaI digestion, and the TT-P_*mntABC*_ and *mntC* PCR products were joined and cloned using Gibson assembly master mix (New England Biolabs) to generate pLH110 (expressing wild-type *mntC*) and pLH107 (expressing the *mntC* allele encoding H50K H123K substitutions). An integrative null vector (containing only TT-P_*mntABC*_) was also constructed by amplification of TT-P_*mntABC*_ from pLH76 with primers oLH671 and oLH672. The resulting fragment was cloned into pLH71 as described above, and the clone was named pLH112. Integrative complementation plasmids were purified from E. coli DC10B (69) and introduced into electrocompetent PFESA0179 *mntC* harboring temperature-sensitive bacteriophage L54a *int* expression vector pLH69 (40). pLH69 was subsequently cured from the resulting transformants, and plasmid integration at the lipase gene, *geh*, was confirmed by PCR. DNA sequencing was used to confirm the integrity of the complementing gene cassette.

### Murine model of sepsis.

Female 9-to-12-week-old CD1 mice (Charles River Laboratories, Inc., Wilmington, MA) were used for virulence studies. S. aureus challenge strains were cultured in TSB medium, and 9 or 10 mice per bacterial strain were inoculated with approximately 1 × 10^8^ CFU via tail vein injection. Survival was monitored for at least 4 days postchallenge. Data were analyzed using GraphPad Prism 6 software (GraphPad Software, Inc., La Jolla, CA). Kaplan-Meier survival curves were plotted, and statistical significance was assessed with log rank (Mantel-Cox) tests. All animal work was performed in strict accordance with approved Institutional Animal Care and Use Committee (IACUC) protocols at an American Association of Laboratory Animal Science (AALAS)-accredited facility (Pfizer, Inc., Pearl River, NY).

10.1128/mSphere.00336-18.1 TEXT S1  Supplemental Materials and Methods. Download TEXT S1, DOCX file, 0.02 MB. Copyright © 2018 Handke et al. 2018 Handke et al. This content is distributed under the terms of the Creative Commons Attribution 4.0 International license.
