# Supplementary figures and images for: MntC-Dependent Manganese Transport Is Essential for Staphylococcus aureus Oxidative Stress Resistance and Virulence
Source: mSphere. 2018 Jul 18;3(4):e00336-18. doi: 10.1128/mSphere.00336-18 (PMC6052334; doi:10.1128/mSphere.00336-18)

Fig. S1.

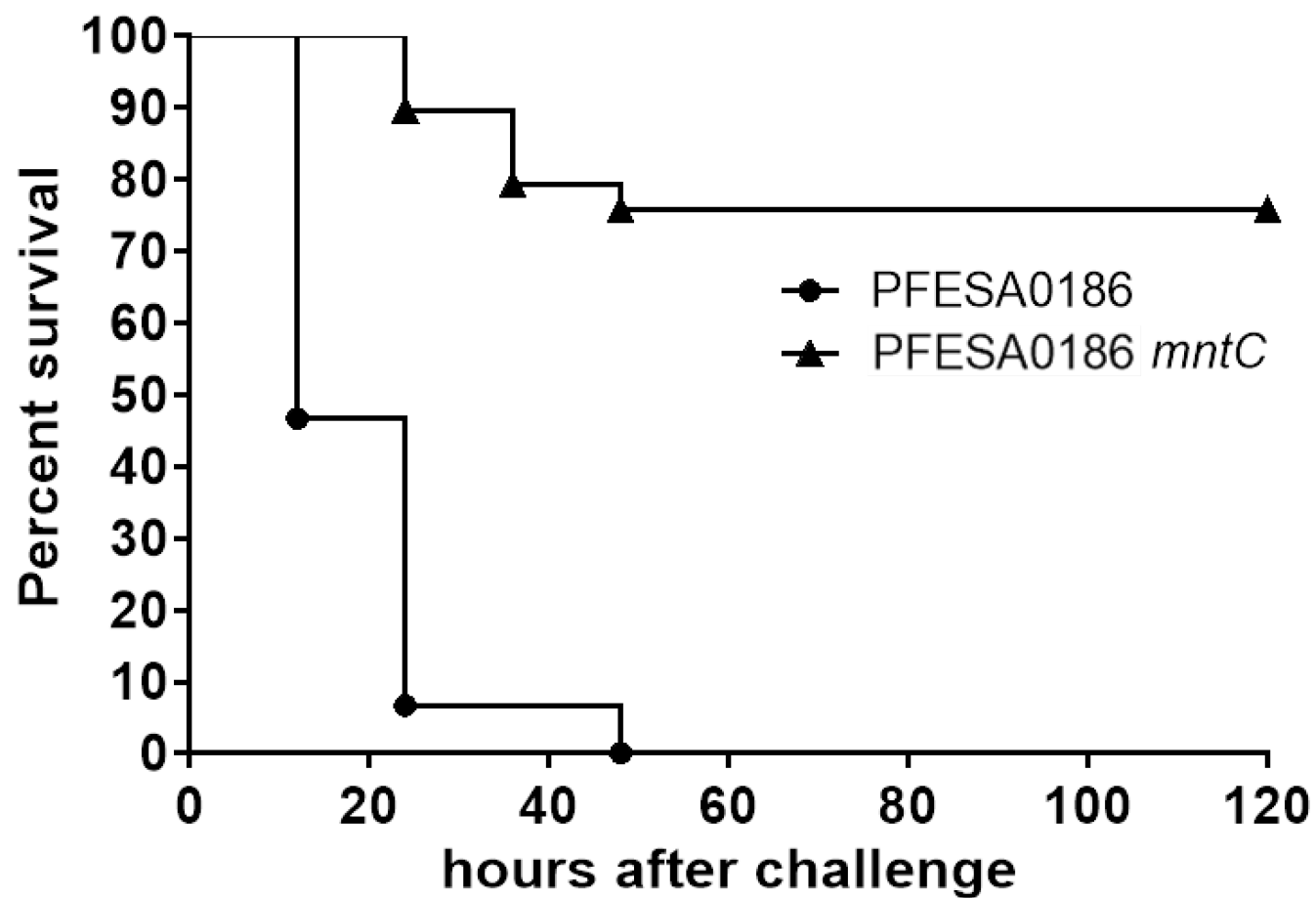

Supplement: FIG S1 [file sph004182591sf1.pdf]

**Fig. S2.**

**A**

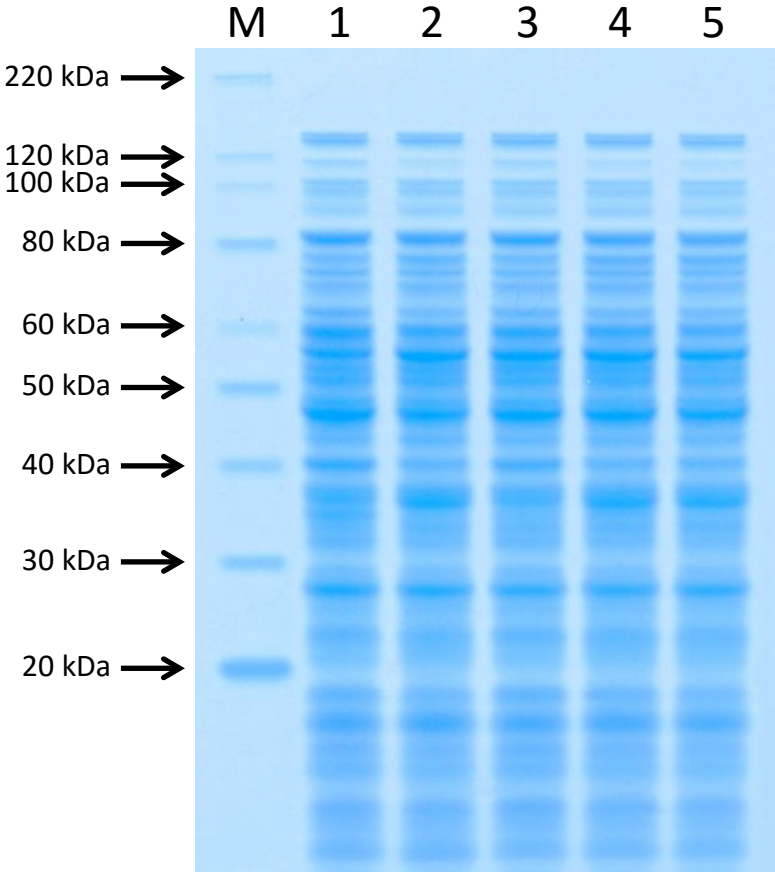

**B**

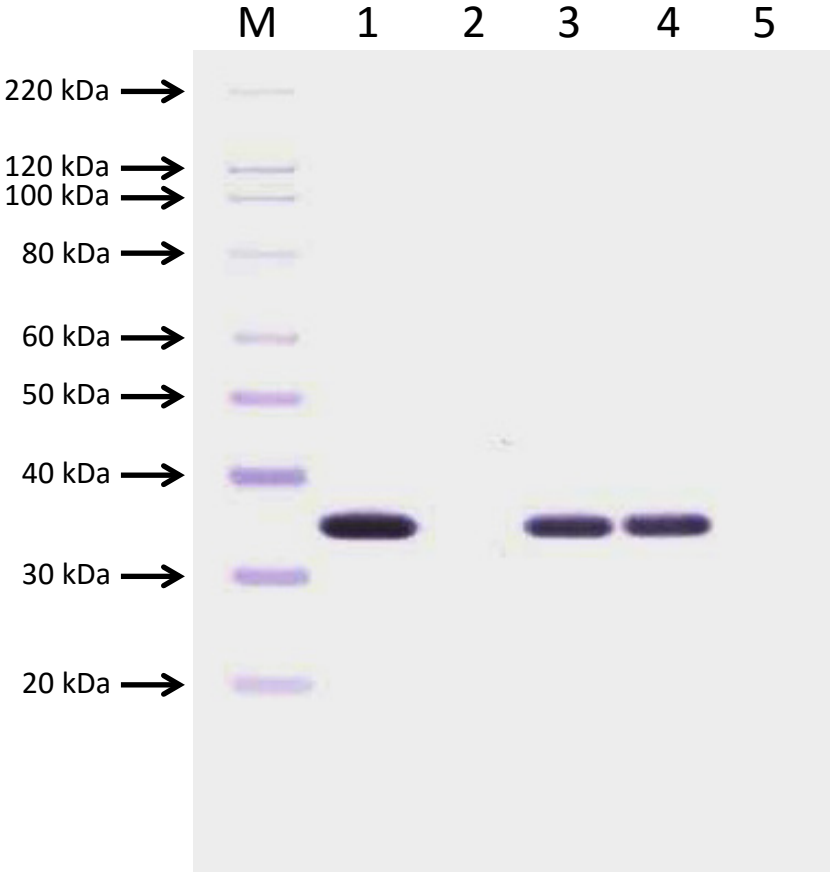

Supplement: FIG S2 [file sph004182591sf2.pdf]
