# Supplementary material for: MntC-Dependent Manganese Transport Is Essential for Staphylococcus aureus Oxidative Stress Resistance and Virulence
Source: mSphere. 2018 Jul 18;3(4):e00336-18. doi: 10.1128/mSphere.00336-18 (PMC6052334; doi:10.1128/mSphere.00336-18)
Supplement: TABLE S1 [file sph004182591st1.docx]

**Supplemental Tables**

**Table S1. Strains and reagents used in the study.**

| PCR Primers: |  |  |
| --- | --- | --- |
| Primer name: | Sequence^a^: |  |
| oLH81 | GCT GCT GGA TCC ACA CTA CGA CAG ATT TGT ACC |  |
| oLH342 | CCA TAA GAA CGG CTC CAA TTA ATT ACC AGC CTG TTC TAA GGC |  |
| oLH343 | GCC TTA GAA CAG GCT GGT AAT TAA TTG GAG CCG TTC TTA TGG |  |
| oLH344 | GGT TTA ACA TCT TTT GAT ACT GCG TCT GGA CTT GAC TCA CTT CC |  |
| oLH345 | GGA AGT GAG TCA AGT CCA GAC GCA GTA TCA AAA GAT GTT AAA CC |  |
| oLH314 | GCT GCT CCC GGG AAC TTC TAG CTT TTC TCT TTC G |  |
| oLH315 | GCT GCT GGA TCC CAT TTG TAA CAA GGT CAT TCG G |  |
| oLH316 | GTA GCA TGT CTC ATT CAA TTA ACA GCG ACT AAT AAC CCA GG |  |
| oLH317 | CCT GGG TTA TTA GTC GCT GTT AAT TGA ATG AGA CAT GCT AC |  |
| oLH318 | GGT CCC ATC AAG TCT TTA TTA CAA AAC TGG TTT AAG CCG AC |  |
| oLH319 | GTC GGC TTA AAC CAG TTT TGT AAT AAA GAC TTG ATG GGA CC |  |
| oLH320 | GCT GCT CCC GGG GAA CTA GGT ATT GAA GAT GAT GG |  |
| oLH321 | GCT GCT GGA TCC GTC GAT TCT TTA CTA TAT CAC GG |  |
| oLH322 | CTT TCT TAT CTT GAT AAT AAG GGA TGA TGA TAG ACA GTA ATA TGG C |  |
| oLH323 | GCC ATA TTA CTG TCT ATC ATC ATC CCT TAT TAT CAA GAT AAG AAA G |  |
| oLH324 | GCA TCA CCA TAA GGT GGA TTA GCC TTC TTC AAC TAA CGG GG |  |
| oLH325 | CCC CGT TAG TTG AAG AAG GCT AAT CCA CCT TAT GGT GAT GC |  |
| oLH326 | GCT GCT CCC GGG ATT GTT CTC TAG CTG CAT ATG G |  |
| oLH355 | GCT GCT GGA TCC ACC ATA TTA TGA TTC TTT AGT AGC |  |
| oLH356 | GCT GCT CCC GGG TGC ATA TGG TAA AGA ATT AAG TGC |  |
| oLH551 | GTA TTG TAC CTG TTG GTC AAG ATC CTA AGG AAT ATG AAG TTA AAC CTA AAG ATA T |  |
| oLH552 | AGA AGG CAA CAA AGA TAA ACA AGA TCC AAA GGC ATG GTT AAG TTT AGA TAA T |  |
| oLH646 | GGT TAG CCT AAA CTT TTA ATT CAC ATT ATT TTT CAC AAA ATT TAC G |  |
| oLH647 | CGA CGT TGT CTC CAC CAA CAT TTT TAG CC |  |
| oLH648 | GGC TAA AAA TGT TGG TGG AGA CAA CGT CG |  |
| oLH615 | GCT GCT CTG CAG TTA TTT CAT GCT TCC GTG TAC AG |  |
| oLH649 | TCG AGC TCG GTA CCC TAT TCT AAA TGC ATA ATA AAT ACT GAT AAC |  |
| oLH650 | TGA AAA ATA ATT GAA TTA AAA GTT TAG GCT AAC C |  |
| oLH651 | ACT TTT AAT TCA ATT ATT TTT CAC AAA ATT TAC GAA TAG AAA G |  |
| oLH652 | CTC TAG AGG ATC CCC TTA TTT CAT GCT TCC GTG TAC |  |
| oLH671 | TCG AGC TCG GTA CCC TAT TCT AAA TGC ATA ATA AAT ACT GAT AAC |  |
| oLH672 | CTC TAG AGG ATC CCC TGA ATT AAA AGT TTA GGC TAA CC |  |
|  |  |  |
| *S. aureus* strains: |  |  |
| Strain name: | Relevant characteristics: | Source or reference: |
| PFESA0179 | Clinical isolate, CC5 | (1) |
| PFESA0186 | Clinical isolate, CC30 | (1) |
| Newman | Template for mntH-1, mntH-2, SA1432-1, and SA1432-2 fragments for *mntH* and SA1432 gene knockout constructs | (2) |
| COL | Template for mntC-1 and mntC-2 fragments for the *mntC* gene knockout construct | (3) |
| RN4220 | Restriction-deficient RN450 | (4) |
| 8325-4 | NCTC8325 cured of three prophage | (5) |
|  |  |  |
| Plasmids: |  |  |
| Plasmid name: | Relevant characteristics: | Source or reference: |
| pGO1 | Template for *aacA-aphD* | (6) |
| pC194 | Template for *cat* | (7) |
| pE194 | Template for *ermC* | (8) |
| pSPT181 | Temperature-sensitive *E. coli*-*S. aureus* shuttle vector | (9) |
| pLH57 | *mntC* knockout vector *- mntC*::*aacA-aphD* cassette cloned into pSPT181 at the *Bam*HI/*Xma*I sites | This study |
| pLH58 | *mntH* knockout vector *- mntH*::*ermC* cassette cloned into pSPT181 at the *Bam*HI/*Xma*I sites | This study |
| pLH59 | SA1432 knockout vector *–* SA1432::*cat* cassette cloned into pSPT181 at the *Bam*HI/*Xma*I sites | This study |
| pLP1215 | Recombinant MntC expression vector | (10) |
| pLH89 | Recombinant MntC H50K H123K expression vector | This study |
| pLH76 | TT-P*_mntABC_*-R*luc* cassette cloned into the integrative vector, pLH71, at the *Eco*RI/*Bam*HI sites. | (1) |
| pLH110 | Integrative complementation vector for expression of wild-type MntC | This study |
| pLH107 | Integrative complementation vector for expression of MntC H50K H123K | This study |
| pLH112 | Integrative complementation null vector | This study |
| pLH69 | pNL9164 with the L54a *int* gene cloned in at the *Sph*I and *Kpn*I sites | (1) |
| pLH71 | *attP*-*tet* cassette cloned into the *Aat*II site in pUC19 | (1) |

^a^ Restriction sites are underlined.

**References**

1. Handke LD, Hawkins JC, Miller AA, Jansen KU, Anderson AS. 2013. Regulation of *Staphylococcus aureus* MntC Expression and Its Role in Response to Oxidative Stress. PLoS One 8:e77874.

2. Duthie ES, Lorenz LL. 1952. Staphylococcal coagulase; mode of action and antigenicity. J Gen Microbiol 6:95-107.

3. Niemeyer DM, Pucci MJ, Thanassi JA, Sharma VK, Archer GL. 1996. Role of *mecA* transcriptional regulation in the phenotypic expression of methicillin resistance in *Staphylococcus aureus*. J Bacteriol 178:5464-71.

4. Kreiswirth BN, Lofdahl S, Betley MJ, O'Reilly M, Schlievert PM, Bergdoll MS, Novick RP. 1983. The toxic shock syndrome exotoxin structural gene is not detectably transmitted by a prophage. Nature 305:709-12.

5. Novick R. 1967. Properties of a cryptic high-frequency transducing phage in *Staphylococcus aureus*. Virology 33:155-66.

6. Archer GL, Coughter JP, Johnston JL. 1986. Plasmid-encoded trimethoprim resistance in staphylococci. Antimicrob Agents Chemother 29:733-40.

7. Horinouchi S, Weisblum B. 1982. Nucleotide sequence and functional map of pC194, a plasmid that specifies inducible chloramphenicol resistance. J Bacteriol 150:815-25.

8. Iordanescu S. 1976. Three distinct plasmids originating in the same *Staphylococcus aureus* strain. Arch Roum Pathol Exp Microbiol 35:111-118.

9. Janzon L, Arvidson S. 1990. The role of the delta-lysin gene (*hld*) in the regulation of virulence genes by the accessory gene regulator (*agr*) in *Staphylococcus aureus*. EMBO J 9:1391-9.

10. Anderson AS, Scully IL, Timofeyeva Y, Murphy E, McNeil LK, Mininni T, Nunez L, Carriere M, Singer C, Dilts DA, Jansen KU. 2012. *Staphylococcus aureus* manganese transport protein C is a highly conserved cell surface protein that elicits protective immunity against *S. aureus* and *Staphylococcus epidermidis*. J Infect Dis 205:1688-96.
