# Supplementary material for: MntC-Dependent Manganese Transport Is Essential for Staphylococcus aureus Oxidative Stress Resistance and Virulence
Source: mSphere. 2018 Jul 18;3(4):e00336-18. doi: 10.1128/mSphere.00336-18 (PMC6052334; doi:10.1128/mSphere.00336-18)
Supplement: TEXT S1 [file sph004182591s1.docx]

**Supplemental Materials and Methods**

***Murine model of sepsis.*** Female 9-12 week old CD1 mice (Charles River Laboratories, Wilmington, MA) were used for virulence studies. *S. aureus* challenge strains were cultured in TSB medium, and nine or ten mice per bacterial strain were inoculated with approximately 1 × 10^8^ CFU via tail vein injection. Survival was monitored for at least four days post-challenge. Data were analyzed using GraphPad Prism 6 software (GraphPad Software, Inc., La Jolla, CA). Kaplan-Meier survival curves were plotted, and statistical significance was assessed with Log-rank (Mantel-Cox) tests. All animal work was performed in strict accordance with approved Institutional Animal Care and Use Committee (IACUC) protocols at an American Association of Laboratory Animal Science (AALAS) accredited facility (Pfizer, Pearl River, NY).

***Western blot hybridization for MntC.*** Cells were grown overnight in TSB-c at 37°C with shaking, and diluted 1:200 the following day in fresh TSB-c. Cells were harvested during mid-exponential phase (OD_600_ = 0.6-0.8), lysed with lysostaphin, and proteins were denatured in SDS-PAGE loading buffer. MagicMark XP Western Protein Standard (ThermoFisher Scientific, Waltham, MA) was used as the protein molecular weight standard. Following electrophoretic separation, proteins were either stained with SimplyBlue SafeStain (ThermoFisher Scientific) or transferred to PVDF membrane (iBlot Transfer Stack, PVDF, ThermoFisher Scientific). The membrane was probed with murine anti-*S. aureus* MntC 305-78-7 monoclonal antibody (1) (at 3 µg/mL) as the primary antibody. Goat anti-mouse IgG (H+L)-AP Conjugate (Bio-Rad, Hercules, CA) at a 1:5,000 dilution was used as the secondary antibody. Western blots were processed using standard protocols (2).

**References**

1. Anderson AS, Scully IL, Timofeyeva Y, Murphy E, McNeil LK, Mininni T, Nunez L, Carriere M, Singer C, Dilts DA, Jansen KU. 2012. *Staphylococcus aureus* manganese transport protein C is a highly conserved cell surface protein that elicits protective immunity against *S. aureus* and *Staphylococcus epidermidis*. J Infect Dis 205:1688-96.

2. Gallagher S, Winston S, Fuller S, Hurrell J. 2008. Immunoblotting and Immunodetection, p 10.8.1-10.8.28, Current Protocols in Molecular Biology, vol 83. John Wiley & Sons, Inc.
